# Supplementary material for: Strategies to Target the Tumor-Associated Macrophages in the Immunosuppressive Microenvironment of Pancreatic Ductal Adenocarcinoma
Source: Cancers (Basel). 2025 Sep 22;17(18):3090. doi: 10.3390/cancers17183090 (PMC12468212; doi:10.3390/cancers17183090)
Supplement: Supplementary file 1 [file cancers-17-03090-s001.zip › cancers-3834301-supplementary.pdf]

Supplemental Table S1. Current status of CAR macrophage studies

Object	Target antigens	Macrophage source	Extracellular/ Intracellular domains	Introduction of CAR	Reference
Glioblastoma	CD19, CD22	J774A.1 Macrophages	Extra: scFv / Intra: Megf10, FcγR, CD3ζ, FcγR + PI3K	Lentivirus	Morrissey, M.A. et al. Elife.2018 [136]
Leukemia, Ovarian cancer	CD19	induced pluripotent stem cells (iPSCs)	Extra: scFv / Intra: CD86 + FcγRI	Lentivirus	Zhang, L. et al. J Hematol Oncol. 2020 [170]
Breast cancer	HER2	Raw264.7 monocyte/ macrophages	Extra: scFv / Intra: CD147	Lentivirus	Zhang, W. et al. Br J Cancer. 2019 [140]
Breast cancer	CCR7	Raw264.7 monocyte/ macrophages	Extra: CCL19 / Intra: TLR2, TLR4, TLR6, MerTK, 4-1BB-CD3ζ	Lentivirus	Niu, Z. et al. J Pathol. 2021 [161]
Ovarian cancer	HER2, mesothelin	Human THP-1 cell line	Extra: scFv / Intra: CD3ζ	chimeric adenoviral (Ad5f35)	Klichinsky, M. et al. Nat Biotechnol. 2020 [138]
Glioblastoma	GPC3, EGFRvIII	induced pluripotent stem cell-derived macrophages(IMACs)	Extra: scFv / Intra: CD3ζ + TIR	Lentivirus	Lei, A. et al. Nat Immunol. 2023
Neuroblastoma	ALK	bone marrow-derived macrophages(BMDMs)	Extra: scFv / Intra:CD3ζ and IFNγ	MPEI: mannose conjugated polyethylenimine nanocarriers containing pcDNA CAR	Kang, M. et al. Adv Mater. 2021
Glioblastoma	HER2	Raw264.7 monocyte/ macrophages	Extra: scFv / Intra:CD3ζ	RP-182 nanoparticles	Gao, L. et al. J Nanobiotechnology. 2023
Hepatocellular	GPC3	Raw264.7 monocyte/ macrophages	Extra: scFv / Intra:CD3ζ	LNP: lipid nanoparticle	Yang, Z et al. J Control Release. 2023
PJI:periprosthetic joint infection	SasA	RAW 264.7 macrophages and bone marrow-derived macrophages	Extra: scFv / Intra:CD3ζ	PNP: peptide nanoparticle	Li, Z. et al. Sci Adv. 2023
Liver fibrosis	uPAR	bone marrow-derived macrophages	Extra: scFv / Intra:CD3ζ	Adenoviruses	Dai, H. et al. J Hepatol. 2024
Leukemia	CD19	induced pluripotent stem cells (iPSCs)	Extra: scFv / Intra:FcRγ+ CD19	Lentivirus	Abdin, S.M. et al. J Immunother Cancer. 2023
Glioblastoma	Glioblastoma	human pluripotent stem cells (hPSCs)	Extra: chlorotoxin / Intra:CD3ζ	Lentivirus	Jin, G. et al. Immunooncol Technol. 2023

Pancreatic cancer	PSCA	induced pluripotent stem cells (iPSCs)	Extra: scFv / Intra:CD3ζ	Lentivirus	Shah, Z. et al. Cell Stem Cell. 2024 [171]
Breast cancer	CD47	Human THP-1 cell line	Extra: scFv / Intra:CD3ζ	Lentivirus	Chuang,S.T. et al. Adv Mater. 2024
Pancreatic cancer	c-Met	Human THP-1 cell line	Extra: scFv / Intra:CD19, CD3ζ	Adenoviruses	Zheng, H. et al. Mol Cancer. 2024 [168]
Pancreatic cancer	c-Met	induced pluripotent stem cells (iPSCs)	Extra: scFv / Intra:FcεRI + CD19	Lentivirus	Hu, L. et al. Mol Med. 2024 [169]
Pancreatic cancer	FAP	Raw264.7 monocyte/ macrophages and bone marrow derived macrophages (BMDMs)	Extra: scFv / Intra:CD3ζ	LNP: lipid nanoparticle	Wang, W. et al. J Control Release. 2025 [167]

Supplemental Table S2. Early phase clinical trials using CAR macrophage.

**Clinical trials ID**	**phase**	**Targeted antigen**	**Condition**	**Sponsor**	**Cell source**	**Status**	**Study objectives**
NCT06224738	Early Phase 1	HER2	Gastric cancer	First People's Hospital of Hangzhou	Autologous	Not yet recruiting	Evaluate the safety and efficacy of human anti-human epidermal growth factor receptor 2(HER2) Chimeric antigen receptor macrophage cells in advanced HER2+ gastric cancer.
NCT06562647	Not Applicable	Mesothelin	Ovarian/pancreatic cancer	Cell Origin Biotech (Hangzhou) Co., Ltd.	Autologous iPSC-derived CAR-Ms	Recruiting	Single-arm, dose-increasing setting study of CAR macrophages in Mesothelin overexpressing solid tumors.
NCT04660929	Phase 1	HER2	Breast and gastric/gastroesophageal junction cancers	Carisma Therapeutics Inc	Adenovirally Transduced Autologous Macrophages	Active, not recruiting	Assess the safety and tolerability of CT-0508 by estimating the frequency and severity of adverse events in subjects with HER2 overexpressing solid tumors.
NCT05007379	Prospective Cohort Study	HER2	Breast cancer	Centre Oscar Lambret	Breast Cancer Patients' Derived Organoids (CARMA)	Unknown status	Collect tumor samples to develop patients' derived organoids to test the antitumor activity of newly developed CAR macrophages.

NCT03608618	Phase 1	Mesothelin	Serous adenocarcinoma, primary peritoneum, or fallopian tube epithelioid or biphasic peritoneal mesothelioma	MaxCyte, Inc.	Autologous Intraperitoneal mRNA-based anti-mesothelin CAR-PBMC MCY-M11	Terminated	Characterize the feasibility, safety and tolerability of MCY-M11 when administered as an intraperitoneal (IP) infusion for 3 weekly doses for women with platinum resistant high grade serous adenocarcinoma of the ovary, primary peritoneum, or fallopian tube, and subjects with peritoneal mesothelioma with recurrence after prior chemotherapy.
